# Rapid Immunoenzyme Assay of Aflatoxin B1 Using Magnetic Nanoparticles

**DOI:** 10.3390/s141121843

**Published:** 2014-11-18

**Authors:** Alexandr E. Urusov, Alina V. Petrakova, Maxim V. Vozniak, Anatoly V. Zherdev, Boris B. Dzantiev

**Affiliations:** 1 A.N. Bach Institute of Biochemistry of the Russian Academy of Sciences, Leninsky Prospect 33, Moscow 119071, Russia; E-Mails: urusov.alexandr@gmail.com (A.E.U.); alina.petrakova@gmail.com (A.V.P.); zherdev@inbi.ras.ru (A.V.Z.); 2 IL Test-Pushchino Ltd., Gruzovaya Street 1g, Pushchino 142290, Moscow Region, Russia; E-Mail: maxin@test-p.ru

**Keywords:** immunoassay, magnetic particles, mycotoxins, aflatoxin

## Abstract

The main limitations of microplate-based enzyme immunoassays are the prolonged incubations necessary to facilitate heterogeneous interactions, the complex matrix and poorly soluble antigens, and the significant sample dilutions often required because of the presence of organic extractants. This study presents the use of antibody immobilization on the surface of magnetic particles to overcome these limitations in the detection of the mycotoxin, aflatoxin B1. Features of the proposed system are a high degree of nanoparticle dispersion and methodologically simple immobilization of the antibodies by adsorption. Reactions between the immobilized antibodies with native and labeled antigens are conducted in solution, thereby reducing the interaction period to 5 min without impairing the analytical outcome. Adsorption of immunoglobulins on the surface of magnetic nanoparticles increases their stability in aqueous-organic media, thus minimizing the degree of sample dilution required. Testing barley and maize extracts demonstrated a limit of aflatoxin B1 detection equal to 20 pg/mL and total assay duration of 20 min. Using this method, only the 3-fold dilution of the initial methanol/water (60/40) extraction mixture in the microplate wells is necessary. The proposed pseudo-homogeneous approach could be applied toward immunodetection of a wide range of compounds.

## 1. Introduction

The enzyme-linked immunosorbent assay (ELISA) in microplate format technique is widely used for the detection of various compounds, and is the most common immunoassay technique with a large number of commercially produced kits available [1,2]. Its main advantages are high sensitivity, ease of use, small sample quantity requirements, and compatibility with simple methods of sample preparation [3,4]. However, traditional ELISA has several drawbacks.

Firstly, ELISA usually takes several hours because of the diffusion-dependent heterogeneous reactions required for the formation of detectable immune complexes [3]. The possibility of reducing the duration of ELISA in some cases by 50 min [5] up to 2 h [6] has been reported, but the optimal reaction time of these interactions have not been analysed in detail.

Secondly, problems arise when the detection compounds are characterized by low solubility in aqueous-saline environments, and therefore require extraction using organic solvents such as methanol, thus causing antibody denaturation [7]. Water-methanol mixtures with a methanol content of 60% or 70% are most often used for mycotoxin extraction. Some researchers have demonstrated the efficiency of 55% methanol and even its 50% (Aflatoxins B1 [AFB1] ELISA Test Kit, Krishgen Biosystems, Los Angeles, CA, USA) or 33% (Total Aflatoxin ELISA Kit, EuroClone SpA, Milan, Italy) content. Other recommended mixtures for the extraction, such as acetonitrile-water, acetonitrile-methanol, methanol-ether, are characterized by similar or higher organic solvent values [8–10]. However, high concentrations of the organic solvent reduce antibody stability for later immunoassay implementation. Thus, loss of antigen-binding properties was described for reaction media containing 20% and even 5% of methanol [11].

To overcome these problems, different approaches are used. An effective solution for reducing ELISA duration is to implement it in pseudo-homogeneous mode, where dispersed carriers coated with immobilized immunoreagents are used for immune interactions and then separated from the reaction mixture for the subsequent detection of carrier-bound enzyme-labelled compounds. Various carriers, such as oppositely charged polyelectrolytes, ultradispersed particles, can be used for this purpose [12,13]. However, manipulation with such carriers for efficient separation could be realized only by the use of additional complicated devices and/or assay stages.

The influence of organic solvents may be excluded by a substantial (twofold or more) dilution of the sample prior to analysis, but this approach causes proportional reduction of the assay sensitivity. Alternatively, more sensitive detection of enzyme labels may be used, for example chemiluminescent detection for peroxidase label instead of common colorimetric approaches [14,15]. These changes also are associated with significant complication of ELISA protocol and instrumentation.

In the given work we propose to use magnetic nanoparticles as solid phase in ELISA. A number of papers have described the application of magnetic nanoparticles in immunoassays of, for example, hormones [16], bacteria [17], allergens [18], proteins [6,19] and viruses [20]. The complexes between the nanoparticles and antibodies are commonly formed by covalent immobilization [21,22]. The dominating approach is to separate target compound by magnetic carriers and then to detect eluted molecules [23–25]. These studies also did not involve the detection of low-solubility antigens and therefore did not consider the question of antibody on particles stability in organic media.

Our proposition is to use the formed complexes of magnetic nanoparticles, immobilized antibodies and bound antigen molecules directly in the ELISA. Increased stability of immobilized antibodies to denaturation will be applied to test samples with relatively high content of methanol. The distinguishing feature of the method proposed here is the use of highly dispersed (average diameter of 10 nm) magnetic carriers and the simple adsorption of antibodies.

In this paper, we describe the realization of an express immunoassay using magnetic nanoparticles for the mycotoxin, aflatoxin B1 (AFB1). Because of multiple toxic effects, AFB1 is one of the priority contaminants for monitoring and control in agricultural products and foodstuffs [26,27]. Nowadays, several commercial ELISA kits for AFB1 are manufactured, e.g., Total Aflatoxin ELISA Kit, EuroClone SpA-LOD 4 ng/g, Milan, Italy; Aflatoxins B1 [AFB1] ELISA Test Kit-LOD 1 ng/g, Krishgen Biosystems, Los Angeles, CA, USA, Total Aflatoxin ELISA Test Kit (Bioo Scientific, Austin, TX, USA-LOD 0.5 ng/g). They allow to find exceeding maximal residue levels for main foodstuffs (official MRLs are from 2 to 12 ng/g), but not enough for special kinds of food, such as baby food with MRL = 0.1 ng/g (Commission Regulation [EU] No 165/2010). Low LODs for AFB1 ELISA may be found in some research papers, such as 10 pg/mL and 90 pg/mL in [14,15], respectively. However, such LODs are achieved through complicated chemiluminescent detection.

Therefore, highly sensitive determination of AFB1 is the demanded task. The results presented here therefore include preparation of magnetic nanoparticles and their non-covalent complexes with antibodies, use of these complexes for rapid determination of aflatoxin B1, and application of this assay for monitoring of plant extracts containing high contents of organic solvents.

## 2. Experimental Section

### 2.1. Materials, Reagents and Equipment

Aflatoxin B1 (Khromresurs, Moscow, Russia), aflatoxin B1-BSA, 3,3′,5,5′-tetramethylbenzidine (TMB), Triton X-100, iron(III) chloride, iron(II) chloride, (Sigma-Aldrich, St. Louis, MO, USA), and bovine serum albumin (BSA; MP Biomedicals, Santa Ana, CA, USA) were used. All other reagents were of analytical grade or higher.

Monoclonal antibodies against AFB1, and AFB1 conjugated with horseradish peroxidase (AFB1-HRP), were provided by IL-TEST Pushchino, Ltd (Pushchino, Moscow Region, Russia). Antibody specificities were confirmed previously [13].

A MagnetoPURE 96 (Chemicell, Berlin, Germany) was used for magnetic separations in 96-well plates and a neodymium magnet 30 × 30 × 30 mm (LLC MAGNET-MSCs, Moscow, Russia) was used for all other magnetic separations.

ELISA were performed with Costar 9018 (Corning, New York, NY, USA) and Medpolymer (St.-Petersburg, Russia) microplates. When conducting ELISA, absorbance of the reaction product was detected with a Zenyth 3100 microplate reader (Anthos Labtec Instruments, Salzburg, Austria).

### 2.2. Microplate ELISA for AFB1

Antibodies against AFB1 were incubated in a microplate for 2 h at 37 °C at a concentration of 1 μg/mL in 100 μL of 50 mM phosphate buffer, pH 7.4, containing 100 mM NaCl (PBS). After four washes with PBS containing 0.05% Triton X-100 (PBST), a solution of AFB1 (50 μL) at concentrations between 5 ng/mL and 0.25 pg/mL in PBST, methanol solution, or plant extract in PBST were added, mixed with 50 μL aflatoxin B1-HRP conjugate (100 ng/mL) and incubated for 5–30 min at 37 °C. The microplate wells were then washed four times with PBST. To determine the peroxidase activity, the substrate solution (0.42 mM TMB and 1.8 mM H_2_O_2_ in a 0.1 M sodium citrate buffer, pH 4.0; 100 μL per well) was injected. After incubation at room temperature for 15 min, the reaction was terminated by the addition of 100 μL of 1 M H_2_SO_4_. The absorbance of the reaction product was read at 450 nm. The plot of the absorbance (***y***) *versus* the antigen concentration in the sample (*x*) was drawn with Origin 7.5 software (Origin Lab, Northampton, MA, USA) using the four-parameter function *y* = (*A* – *D*)/(1 + (*x/c*)*^B^*) + *D*. The analytical characteristics of the system were determined based on the resulting function, as described in [28,29].

### 2.3. Synthesis of Magnetic Nanoparticles (MNPs)

This was conducted according to [30,31] with some modifications. An aqueous 0.5% solution of iron salts (II) and (III) in a molar ratio (III):(II) of 2:1 was prepared. A 30% ammonia hydrate solution was added dropwise to a concentration of 8%. After incubation for 15 min at room temperature with thorough mixing, the particles formed were collected with a magnet, and after removal of the supernatant were resuspended in bidistilled water and washed five times with excess distilled water. Literature data state dominating Fe_2_O_3_ in the product of this aerobic synthesis of iron oxide particles [32]. The resulting suspension of MNPs was stored at 4 °C. The obtained preparation did not precipitated for at least three months.

To determine the concentration of the obtained particles, they were washed five times with bi-distilled water and dried in Petri dishes overnight at 36 °C. The difference of the weight for the empty Petri dish and the dish with dried preparations indicates the mass of particles and their content in the initial solution. Characterization of the nanoparticles by transmission electron microscopy is presented in the Supplementary Materials, Section 1.

### 2.4. Immobilization of Antibodies on Magnetic Nanoparticles

MNPs (500 μL) in PBS at 3 mg/mL were mixed with a solution of anti-AFB1 antibodies (2 mg/mL) to obtain a final antibody concentration of 8–70 μg/mL. The mixture was incubated for 30 min with vigorous stirring. MNPs were collected with a magnet and washed three times with PBS. The resulting suspension was stored at 4 °C.

### 2.5. Preparation of Plant Extracts

Milled grains were mixed with an extraction solution (60% methanol, 40% water) at a ratio of 1:5, and incubated with gentle stirring at room temperature for 1 day (in accordance with [33], with modifications). After centrifugation, the supernatant was collected and stored at 4 °C. The extracts were analyzed by HPLC according to [34] and no aflatoxin B1 was detected.

### 2.6. ELISA for AFB1 Using MNP

AFB1 (50 μL) was added to the microplate wells at several dilutions between 5 ng/mL and 0.25 pg/mL in PBST containing 0.1% BSA and supplemented with varying concentrations of methanol (20%–70%). Alternatively, instead of pure AFB1, plant extracts were spiked with varying concentrations of AFB1 (0.2–5000 pg/mL in a final volume of 50 μL containing 60% methanol) were added. Then, 50 μL AFB1-HRP conjugate (600 ng/mL in PBST with 0.1% BSA) were added. The resulting solution was stirred for 10 s and 50 μL of the MNP-antibody conjugate at 90 μg/mL (based on the MNP concentration) in PBST with 0.1% BSA were added. The incubation was performed at room temperature with stirring, varying in duration between 5 and 30 min. The MNPs were then collected by magnet and washed four times with 100 μL of 50 mM phosphate buffer, pH 7.4, containing 100 mM NaCl and 0.05% Triton X-100 (PBST) with 0.1% BSA. The formed immune complexes were detected by peroxidase reaction. The substrate solution (0.42 mM TMB and 1.8 mM H_2_O_2_ in a 0.1 M sodium citrate buffer, pH 4.0; 100 μL per well) was injected. After incubation at room temperature for 15 min, the reaction was terminated by the addition of 100 μL of 1 M H_2_SO_4_. The absorbance of the reaction product was read at 450 nm.

## 3. Results and Discussion

### 3.1. Synthesis of Magnetic Nanoparticles and Their Conjugates with Anti-AFB1 Antibodies

The magnetic nanoparticles were obtained by a co-precipitation technique. Their size was determined by transmission electron microscopy (see Supplementary Materials, Section 1). The average particle diameter was 9.1 ± 3.2 nm, the shape is close to spherical (ratio of axes in the range 1.0–1.3) and single, non-aggregated particles prevailed in the preparation. Note that previously published studies on MNP-based ELISA all used substantially larger carriers with diameters of 0.3–3 μm [35–37]. The use of magnetic particles with a small size increases the total surface area for contacting with the analyte and also enhances the stability of the suspension.

Physical adsorption was used for the conjugation. This approach was first applied to prepare conjugates of magnetic particles with antibodies. However, it used widely to obtain preparations for immunoassay. For example, adsorption of antibodies on the surface of gold nanoparticles is a prevalent practice in immunochromatography [38]. The advantage of adsorption is its methodological simplicity and exclusions of additional influence on protein reagents.

The concentration of anti-AFB1 antibodies was chosen to reach saturation of the adsorption binding sites on the nanoparticles surface. For this purpose quantity of non-bound antibodies were controlled for different added concentrations as described in [39]. The selected protocol is the mixing of 500 μL of MNPs (3 mg/mL) and 16 μL of anti-AFB1 antibodies (2 mg/mL). Final concentration of the antibodies during the synthesis is 62 μg/mL. Its further growth did not cause increase in quantity of adsorbed antibodies.

Our preparations obtained by the physical adsorption were found to be stable and reproducible. The five conjugates did not demonstrate any reliable differences. Storage for at least three months did not cause reduction of antigen-binding activity.

### 3.2. Principle of the Assay

**Figure 1.**
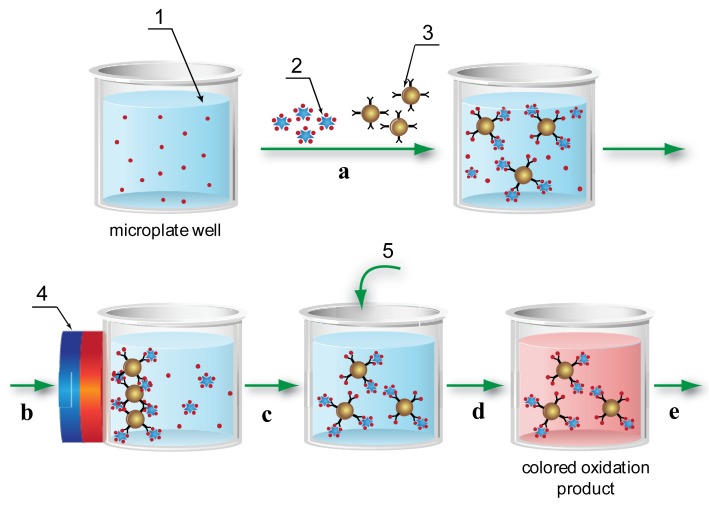
Scheme of ELISA method using magnetic nanoparticles. 1—sample containing AFB1; 2—AFB1-peroxidase conjugate; 3—MNP conjugate with antibodies against AFB1; 4—magnet; and 5—peroxidase substrate. Steps a–e are described in the article.

Using the MNPs as the solid phase allowed a significantly increased surface area for the immobilization of the reactants (see Section 2 of the Supplementary Material) and their uniform distribution throughout the whole volume of the reaction medium, thereby eliminating the diffusion limitations of traditional ELISA. The application of a magnetic field separated the reactants simply and rapidly, and facilitated the wash steps that are also required in traditional microplate-based ELISA. Using these advantages, the following MNP-based immunoassay scheme was developed and implemented in ELISA microplate wells (Figure 1).

Step a: Free AFB1 contained in the test sample competes with peroxidase-labeled AFB1 for binding sites on the antibodies adsorbed on the MNP surface.
Step b: A magnet separated MNPs from unreacted components.
Step c: MNPs are washed and, by removing the magnetic field, returned into solution.
Step d: The peroxidase substrate is added to the MNP suspension.
Step e: Absorbance of the peroxidase reaction product is recorded; this reflects by inverse proportion the AFB1 content in the sample.

It should be noted that for the small size of the obtained MNPs and temperature regime of the assay the obtained MNPs should be superparamagnetic ones [40]. This fact could be important for work with different matrixes. However, in our case these properties do not influence the assay characteristics, such as efficient separation and redissolving without any visible changes of the suspension.

Under the selected ELISA protocol the preparation of MNPs is relatively diluted. Its concentration in the microplate wells is 10 μg/mL during the competitive stage and 15 μg/mL during the enzymatic reaction. Thus, the magnetite particles with density near 5 g/mL takes only 0.2%–0.3% of the reaction mixture volume, and average distance between them is much more that their own diameters. These features of the analytical system exclude magnetic interactions (dipolar coupling) between the particles (the calculations of [41] state their significance for the systems with the ratio of the average distance and the diameter not more that 1.5).

### 3.3. Preparation and Characterization of Assay Reagents

**Figure 2.**
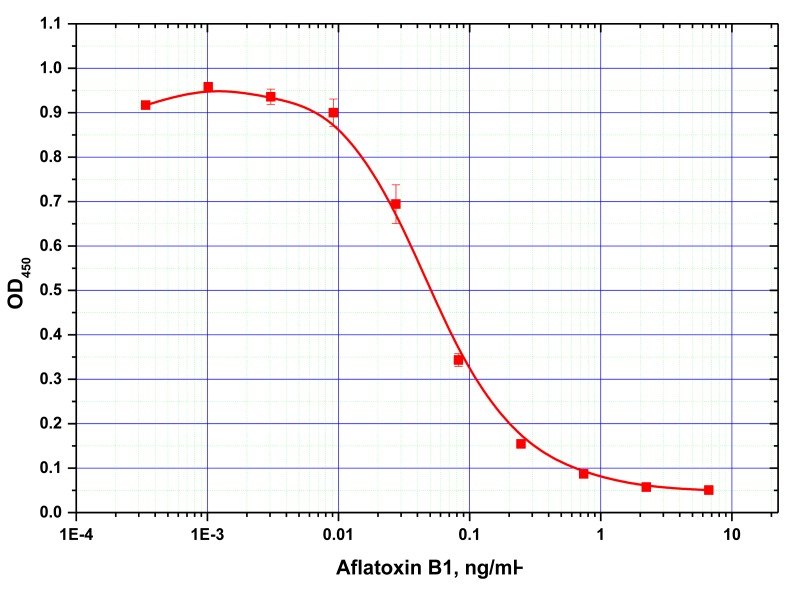
Calibration curve of conventional microplate ELISA for AFB1 detection. A limit of detection of 15 pg/mL was determined. The measurements were performed in triplicates.

Antibodies against AFB1 were first characterized by conventional microplate ELISA. For the selected ratio of the reagents found to be optimal for ELISA (see Experimental Section), the detection limit for AFB1 was determined at 15 pg/mL (Figure 2).

Physical adsorption was used for the preparation of MNP-antibody conjugates. This distinguishes the proposed methodology from traditional methods that use covalent immobilization [35–37]. The optimum MNP/antibody ratio was chosen based on preliminary experiments. Concentration dependence of antibody binding to the MNPs was observed, based on comparisons of the initial antibody concentrations applied and the concentrations of unbound antibody remaining in the supernatants following binding. An antibody concentration of 70 μg/mL was found to be sufficient for ensuring the saturation of sorption sites on the surface of the MNPs. This concentration was therefore chosen for all future conjugate synthesis.

### 3.4. Optimizing the Duration of the Immune Interactions and Development of MNP-Based ELISA

Preselection of the reagent concentrations for the immunoassay was performed under equilibrium conditions, using 1 h incubation at Step A. Varying the concentration of the MNP-antibody and AFB1-HRP conjugates, the assay regime was determined that achieved the minimum detection limit for AFB1 with sufficient signal intensity (the resulting optical density of the peroxidase reaction product was ∼1.0), where low binding of the label in the presence of excess competitor (free AFB1 in the sample) was clearly distinguishable (resulting OD < 0.1). These optimum concentrations were determined at 30 μg/mL for the MNP-antibody conjugate (based on MNP weight) and 600 ng/mL for the AFB1-HRP conjugate.

Note that for this assay protocol the total surface area of the MNPs combined was 6 cm^2^ (see Section 2 of the Supplementary Material), which considerably exceeds the binding area of an ELISA microplate well (0.32 cm^2^, according to Costar data). This difference, combined with the uniform distribution of the MNPs throughout the reaction volume, provides the potential for significant reductions in the time required for analytic immune interactions.

**Figure 3.**
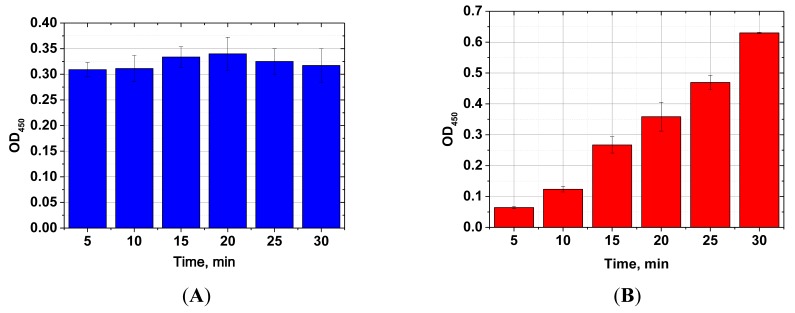
Effect of incubation period on the signal intensity of MNP-based ELISA for AFB1 detection. The AFB1 concentration was 60 pg/mL. No significant effect was observed and 5-min incubation was sufficient for near complete interaction (**A**). Effect of AFB1 and MNP-antibody incubation period on the signal intensity of microplate ELISA for AFB1 detection. No signal plateau was observed over the tested range (**B**). The measurements were performed in triplicates.

**Figure 4.**
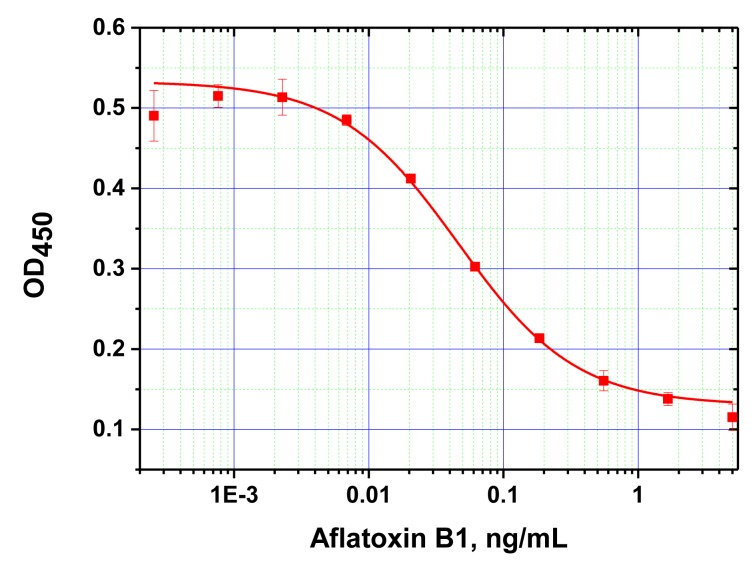
Calibration curve of MNP-based ELISA for AFB1 detection using 5-min incubation period for immune interaction. A limit of detection of 10 pg/mL was obtained. The measurements were performed in triplicates.

Next, ELISA assays with varying Step A duration from 5 to 30 min were compared. As the results in Figure 3 show, the signal intensities (levels achieved in the absence of the competitor) differed by less than 10% across the time intervals tested. Thus, even 5 min incubation was sufficient for near complete immune interaction. Additional reduction of Step A was impractical because a higher degree of error would have been introduced as a result of the inherent variability in the time required for dispensing the reagents into different wells of the microplate. The limit of detection achieved by our MNP-based ELISA was 10 pg/mL (Figure 4), comparable to that obtained using microplate-based ELISA. The working range of the quantitative detection of AFB1 using our method was 10–300 pg/mL.

Note that in the previously described applications of MNPs in ELISA, the duration of the immune interaction required was between 10 and 60 min [35–37,42–44]. For example, in the reaction time studies of the MNP-antibody conjugate interaction with blood cells performed by Waseem *et al*. [44], the authors found that the reaction only reached equilibrium after 20 min. The improved interaction rate observed for our system may be a consequence of the smaller sizes of the antigen and the magnetic nanoparticles facilitating faster diffusion rates.

**Figure 5.**
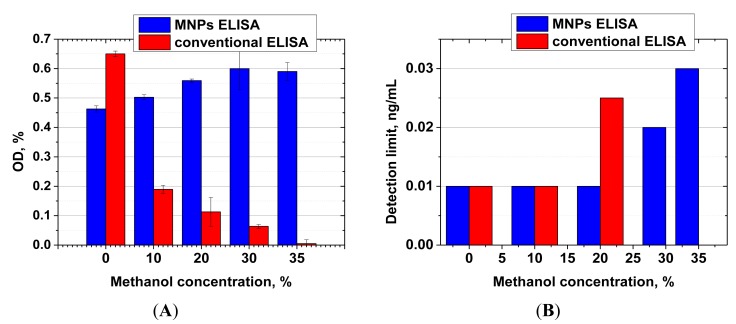
Influence of methanol content on signal intensity (**A**) and limit of AFB1 detection for ELISA (**B**) for MNP-based ELISA (green bars) and microplate-based ELISA (red bars). For microplate ELISA, ≥30% methanol content produced insufficient label binding for reliable competitive detection of AFB1, whereas MNP-based ELISA was still reliable. The measurements were performed in triplicates.

For comparison, Figure 5 presents the reaction time study of the same immune interaction using conventional microplate ELISA. Increased signal was observed with increasing incubation periods, and no signal plateau was reached, even at the maximum incubation period tested (30 min). This illustrates the significant advantages of the proposed system over conventional ELISA for development of an express immunoassay.

### 3.5. The Effect of Methanol on Immune Interactions in MNP- and Microplate-Based ELISA

Both ELISA formats (antibody immobilization on MNPs and on polystyrene microwells) were compared in terms of their stability in the presence of methanol. Figure 5 shows signal intensities and limits of AFB1 detection observed at various concentrations of methanol in the reaction.

For the MNP-based ELISA, the presence of methanol at concentrations up to 20% not only had no negative effect on assay sensitivity, but actually led to an increase in signal intensity. The observed effect may be explained by the fact that the immobilization of antibodies on the MNP surface leads to stabilization of their structure, which prevents denaturation [45,46]. AFB1 is also well dispersed in aqueous-organic mixtures, while in a purely aqueous medium it forms aggregates [47] because of poor solubility, thus reducing the effective concentration of the mycotoxin in the medium and deteriorating the sensitivity of the assay.

For the microplate ELISA, the negative effect of methanol [48] was apparent at much lower concentrations, and at a concentration of 30% methanol the quantity of the formed immune complexes was already insufficient for reliable quantitative detection of the competitive binding of AFB1 present in samples.

These results are consistent with the literature. Melnikova *et al*. showed decreased affinity of the antibody to ferritin already at 5% methanol content, whereas 20% methanol caused a 3.5-fold decrease in the binding constant [11,49]. Wang *et al*. prepared 20-fold dilutions of methanol extracts of corn prior to immunoassay to avoid the negative influence of methanol on the antibody stability [36]. Thus, their reactions were performed in a medium containing only 3.5% methanol, with a corresponding loss in sensitivity. High tolerance of immunoassays toward organics has also been described by He *et al*. [50]. They implemented ELISA detection of AFB1 in the presence of 40% methanol, with the use of recombinant mini-antibodies (nanobodies) from alpaca that are known to be significantly more stable compared with the full-length antibodies [51]. These antibodies are however difficult to source compared with traditional antibodies. Furthermore, their affinities are usually much lower than that of full-length antibodies. Thus, in the He *et al*. study, the detectable range for AFB1 varied between 0.117–5.676 ng/mL. These parameters are lower by one order in comparison with our analytical system.

### 3.6. Application of MNP-Based ELISA for Determining AFB1 in Plant Extracts

**Figure 6.**
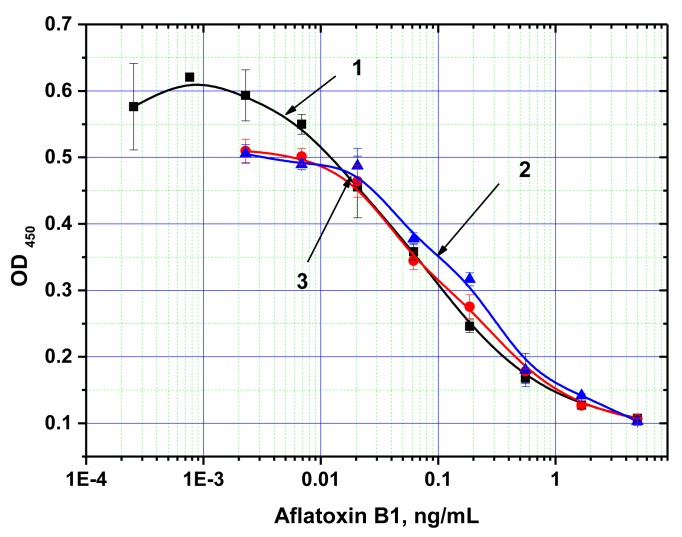
Concentration dependence of AFB1 detection when using MNP-based ELISA. A solution of methanol in buffer (1) and extracts of barley (2) and maize (3) were spiked with known concentrations of AFB1. The measurements were performed in triplicates.

The developed analytical system was then tested for AFB1 detection in real plant extracts using the “added-found” method; *i.e.*, spiking unclarified, crude extracts of maize and barley determined to be mycotoxin free based on previous HPLC analysis, with various known concentrations of AFB1. Adding the extract prepared in the standard way to the MNP-antibody and AFB1-HRP conjugates produced a final methanol content of 20% in the reaction medium, which, as noted above (see Figure 6), did not influence the immune interactions with the antibodies immobilized on the MNP surface. Figure 6 shows the concentration dependence of AFB1 detection using this system.

The working range for AFB1 detection was determined at 0.02–1 ng/mL (0.1–6.0 ng/g of product), and the degree of AFB1 recovery was 79%–82%. According to European Commission regulations (Commission Regulation [EU] No 165/2010), the maximum permissible level of AFB1 in different foodstuffs varies from 2 to 12 ng/g, and reaches 0.1 ng/g for baby foods.

## 4. Conclusions

The ELISA method developed here using magnetic carriers allowed the determination of the mycotoxin aflatoxin B1 in environments with a high content of organic extractant. The specific features of the method are application of small (∼10 nm) magnetic nanoparticles and stabilization of antibodies by adsorption immobilization. The formed complexes of nanoparticles, immobilized antibodies and bound antigen are used in ELISA directly, without elution stages. The separation of the formed immune complexes by applying a magnetic field provided a significant reduction in the incubation period required during the competitive interaction stage of the ELISA, to as little as 5 min. The immobilized antibodies were found to be more stable to methanol, thus allowing to minimize dilution of tested extracts in the course of ELISA. The developed method is characterized a very low LOD (20 pg/mL), which allows practical monitoring and control of aflatoxin B1 levels to below the maximum permissible level in various agricultural products. The universal nature of the proposed method allows its consideration as an effective means of express testing for various contaminants, especially those characterized by low solubility in water-saline media.

## Supplementary Material
