# Supplementary material for: Rapid Immunoenzyme Assay of Aflatoxin B1 Using Magnetic Nanoparticles
Source: Sensors (Basel). 2014 Nov 18;14(11):21843–57. doi: 10.3390/s141121843 (PMC4279564; doi:10.3390/s141121843)
Supplement: Supplementary file 1 [file sensors-14-21843-s001.pdf]

OPEN ACCESS

sensors

ISSN 1424-8220

www.mdpi.com/journal/sensors

Supplementary Information

Rapid Immunoenzyme Assay of Aflatoxin B1 Using Magnetic Nanoparticles. *Sensors* 2014, *14*, 21843-21857

Alexandr E. Urusov [1,†], Alina V. Petrakova [1,†], Maxim V. Vozniak [2], Anatoly V. Zherdev [1] and Boris B. Dzantiev [1,*]

[1] A.N. Bach Institute of Biochemistry of the Russian Academy of Sciences, Leninsky Prospect 33, Moscow 119071, Russia; E-Mails: urusov.alexandr@gmail.com (A.E.U.); alina.petrakova@gmail.com (A.V.P.); zherdev@inbi.ras.ru (A.V.Z.)

[2] IL Test-Pushchino Ltd., Gruzovaya Street 1g, Pushchino 142290, Moscow Region, Russia; E-Mail: maxin@test-p.ru

† These authors contributed equally to this work.

* Author to whom correspondence should be addressed: E-Mail: dzantiev@inbi.ras.ru; Tel.: +7-495-954-31-42; Fax: +7-495-954-28-04.

1. Characterisation of Magnetic Nanoparticles

Transmission Electron Microscopy

Preparations of magnetic nanoparticles were applied to 300-mesh grids (Pelco International, USA) coated with a support film of poly(vinyl formal) dissolved in chloroform. The images were obtained with a JEM CX-100 electron microscope (JEOL, Akishima, Tokyo, Japan) operating at 80 kV. The digital images were analysed with the Image Tool program (University of Texas Health Science Center, San Antonio, TX, USA) (Figure S1).

Figure S1. Study of magnetic nanoparticles: fragment of microphotography (**A**) and size distribution based on the data of transmission electron microscopy (**B**).

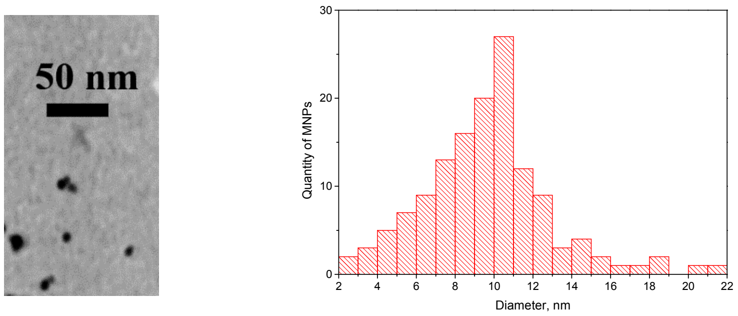

(**A**) (**B**)

2. Calculation of Surface Area for Immune Interaction in the Course of the Proposed MNPs-Based ELISA

The reaction mixture during the competitive stage of the ELISA has volume of 150 µL and contains MNP-antibody conjugate with concentration (by MNP) 30 µg/mL. Thus, the reaction mixture contains 4.5 µg of MNPs. One MNP with diameter 9 nm has volume ~380 nm^3 and surface area ~255 nm^2.

As well as magnetite density is near 5 g/mL (5×10^6 g per m^3), the weight of one nanoparticle is $380 \times 10^{-27} \times 5 \times 10^6$ g = 1.9×10^{-18} g = 1.9×10^{-12} µg. Thus, the reaction mixture contains 2.4×10^{12} particles with total surface $255 \times 10^{-18} \times 2.4 \times 10^{12}$ m^2 = 6 cm^2.

© 2014 by the authors; licensee MDPI, Basel, Switzerland. This article is an open access article distributed under the terms and conditions of the Creative Commons Attribution license (http://creativecommons.org/licenses/by/4.0/).
